# RIPK1 protects from TNF-*α*-mediated liver damage during hepatitis

**DOI:** 10.1038/cddis.2016.362

**Published:** 2016-11-10

**Authors:** Aveline Filliol, Claire Piquet-Pellorce, Jacques Le Seyec, Muhammad Farooq, Valentine Genet, Catherine Lucas-Clerc, John Bertin, Peter J Gough, Marie-Thérèse Dimanche-Boitrel, Peter Vandenabeele, Mathieu JM Bertrand, Michel Samson

**Affiliations:** 1 Institut National de la Santé et de la Recherche Médicale (Inserm), U1085, Institut de Recherche Santé Environnement et Travail (IRSET), Rennes, France; 2 Université de Rennes 1, Rennes, France; 3 Structure Fédérative BioSit UMS 3480 CNRS-US18 Inserm, Rennes, France; 4 Service de Biochimie CHU Rennes, Université de Rennes 1, Rennes, France; 5 Pattern Recognition Receptor Discovery Performance Unit, Immuno-inflammation Therapeutic Area, GlaxoSmithKline, Collegeville, PA, USA; 6 Inflammation Research Center, VIB, Zwijnaarde-Ghent, Belgium; 7 Department of Biomedical Molecular Biology, Ghent University, Zwijnaarde-Ghent, Belgium

## Abstract

Cell death of hepatocytes is a prominent characteristic in the pathogenesis of liver disease, while hepatolysis is a starting point of inflammation in hepatitis and loss of hepatic function. However, the precise molecular mechanisms of hepatocyte cell death, the role of the cytokines of hepatic microenvironment and the involvement of intracellular kinases, remain unclear. Tumor necrosis factor alpha (TNF-*α*) is a key cytokine involved in cell death or survival pathways and the role of RIPK1 has been associated to the TNF*-α*-dependent signaling pathway. We took advantage of two different deficient mouse lines, the RIPK1 kinase dead knock-in mice (Ripk1^K45A^) and the conditional knockout mice lacking RIPK1 only in liver parenchymal cells (Ripk1^LPC-KO^), to characterize the role of RIPK1 and TNF-*α* in hepatitis induced by concanavalin A (ConA). Our results show that RIPK1 is dispensable for liver homeostasis under steady-state conditions but in contrast, RIPK1 kinase activity contributes to caspase-independent cell death induction following ConA injection and RIPK1 also serves as a scaffold, protecting hepatocytes from massive apoptotic cell death in this model. In the Ripk1^LPC-KO^ mice challenged with ConA, TNF-*α* triggers apoptosis, responsible for the observed severe hepatitis. Mechanism potentially involves both TNF-independent canonical NF-*κ*B activation, as well as TNF-dependent, but canonical NF-*κ*B-independent mechanisms. In conclusion, our results suggest that RIPK1 kinase activity is a pertinent therapeutic target to protect liver against excessive cell death in liver diseases.

Contemporary liver diseases result from chronic conditions, such as chronic viral hepatitis, nonalcoholic fatty liver hepatitis (NASH) and alcoholic liver hepatitis.^1^ The global pandemic of chronic viral hepatitis (hepatitis B and C virus infections) affects a significant proportion of the world population, currently estimated at around 500 million people.^2^ In parallel, the prevalence of NASH rises especially in Western countries because of lifestyle evolution. Untreated, chronic hepatitis predispose to the development of cirrhosis and hepatocellular carcinoma (HCC).^3^ The progression of these diseases is triggered by hepatocyte death,^3, 4^ and the cell death processes are starting to emerge. Immune cells, including liver resident macrophages (Kupffer cells) or infiltrating natural killer (NK) cells and T cells (natural killer T (NKT) and T lymphocytes), produce molecules that induce hepatic parenchyma damage. Immune cells secrete or expressed at their surface tumor necrosis factor alpha (TNF-*α*), FAS ligand (FasL) and TNF-related apoptosis-inducing ligand (TRAIL). These ligands induce cell death by engaging their respective receptors (TNFR, Fas and death receptors 4 and 5 (DR4, DR5)) present at the surface of hepatocytes.^5^ Receptor interacting protein kinase 1 (RIPK1) is a key protein regulating signaling downstream of these DRs, and is best characterized for its roles downstream of TNFR1.^6^ Clinical studies have underlined the crucial role of TNF-*α* in several liver diseases. Indeed, serum TNF-*α* levels increase in patients with fulminant hepatic failure^7^ and is correlated with poor prognostic.^8^ TNF-*α* is a master pro-inflammatory cytokine that binds TNFR1 and TNFR2, but most of its biological activities have been associated with TNFR1 signaling. Downstream of TNFR1, RIPK1 functions as a signaling node driving cell survival as well as caspase-8-dependent apoptosis or RIPK3/mixed lineage kinase domain-like pseudokinase (MLKL)-dependent necroptosis.^9^ These opposed cellular fates are regulated by two different faces of RIPK1, it functions as a scaffold to promote cell survival, in part via NF-*κ*B activation, and as a kinase to induce cell death.^6^ The perinatal lethality of RIPK1-deficient mice has long hampered the *in vivo* study of the dual faces of RIPK1.^10^ Recently, the publication of viable and healthy RIPK1 kinase dead knock-in mice (*Ripk1*^K45A^) revealed that only RIPK1 scaffolding function, and not its kinase activity, is crucial for homeostasis and viability.^11^ The generation of conditional RIPK1-deficient mouse lines also demonstrated the crucial pro-survival role of RIPK1 in intestinal and epidermal epithelia and in hematopoietic cells.^12, 13, 14^

RIPK1 functions in the liver, especially under pathological conditions, have so far remained unclear. The hepatic inflammation induced in mice by concanavalin A (ConA) injection reflects the overall physiopathological conditions observed in human viral or autoimmune hepatitis.^15^ In this model, the combined occurrence of usual cytotoxic effectors (perforin, granzyme B, TNF-*α*, FasL and TRAIL) triggers hepatocyte death and takes part in hepatitis development.^16^ Indeed, artificial impairment of one effector, or of its cognate receptor, is sufficient to prevent hepatitis in ConA-challenged animals.^5, 17, 18, 19^ Data suggest implication of RIPK1 kinase activity in the DR-induced death of hepatocytes in this model. Indeed, chemical inhibition of RIPK1 seems to protect the organ from ConA-induced T-cell-mediated injuries.^20, 21, 22^ However, genetic evidence for the implication of RIPK1 kinase activity in the death process is still lacking. In addition, whether RIPK1 also functions as a scaffold maintaining liver homeostasis under physiological condition and/or protecting hepatocytes from death under challenged conditions is currently unknown. Thus, to characterize the role of RIPK1 in hepatitis, we took advantage of two genetically modified mouse lines, the RIPK1 kinase dead knock-in mice (*Ripk1*^K45A^) and the conditional knockout mice lacking RIPK1 only in liver parenchymal cells (*Ripk1*^LPC-KO^).

## Results

### RIPK1 is dispensable for liver homeostasis under steady-state conditions

**Figure 1:**
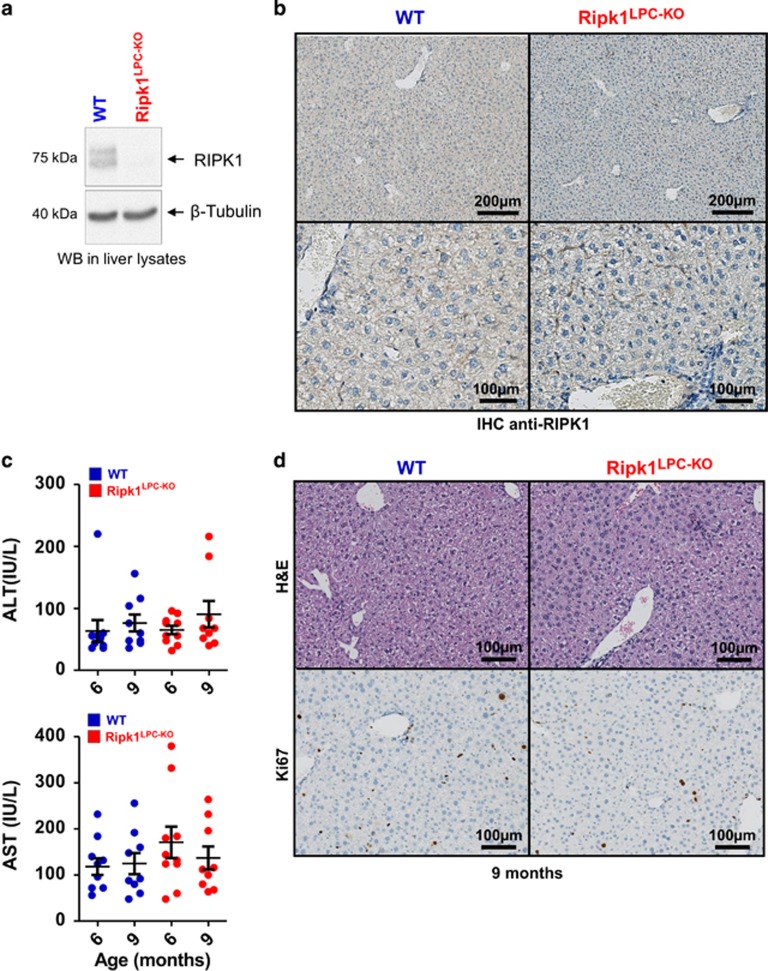
RIPK1 is dispensable for liver homeostasis under steady-state conditions. (**a**) Western blot analysis of RIPK1 and *β*-tubulin in protein extracts issued from the livers of WT and *Ripk1*^LPC-KO^ mice. (**b**) Pictures of liver tissue sections analyzed by IHC for RIPK1 issued from WT and *Ripk1*^LPC-KO^ mice challenged with PBS. (**c**) Levels of serum ALT and AST in unchallenged animals aged 6 and 9 months. (**d**) Pictures of liver tissue sections, stained by H&E or analyzed by IHC for Ki67, issued from WT and *Ripk1*^LPC-KO^ mice aged of 9 months

In order to evaluate the role of RIPK1 in liver homeostasis, we generated mice specifically deficient for RIPK1 in liver parenchymal cell (hepatocytes and cholangiocytes) (*Ripk1*^LPC-KO^) by crossing RIPK1 conditional mice (*Ripk1*^fl/fl^)^12^ with transgenic mice expressing the Cre recombinase under the control of the mouse albumin regulatory elements and *α*-fetoprotein enhancers (Alfp-Cre).^23, 24^ *Ripk1*^LPC-KO^ mice were viable and reached adulthood without morphological liver alteration. Western blot and IHC analyses confirmed RIPK1 expression in the liver parenchymal cells of the *Ripk1*^fl/fl^ wild-type (WT) animals but not of the *RipK1*^LPC-KO^ littermates (Figures 1a and b). Monitoring of *RipK1*^LPC-KO^ mice over a period 9 months did not revealed occurrence of any obvious abnormality. Even if liver histology of mature *Ripk1*^LPC-KO^ mice revealed few infiltrated immune cells, no inflammation settled. Indeed, serum transaminase (aspartate amino-transferase (AST) and alanine amino-transferase (ALT)) levels remained stable and similar to those of WT littermates (Figure 1c). No changes in the expression levels of inflammatory cytokines (TNF-*α*, IFN-*γ* and IL-6) were similarly detected (Supplementary Figure 2A). Finally, Ki67 labeling of liver tissues did not show any modification in basal cell proliferation rate between the two genotypes (Figure 1d). Together, these results showed that, contrary to its role in other tissues,^12, 13^ RIPK1 is dispensable for proper liver development and homeostasis.

### RIPK1 has pro-cell death and anti-apoptotic functions in hepatocyte during ConA-mediated hepatitis

**Figure 2:**
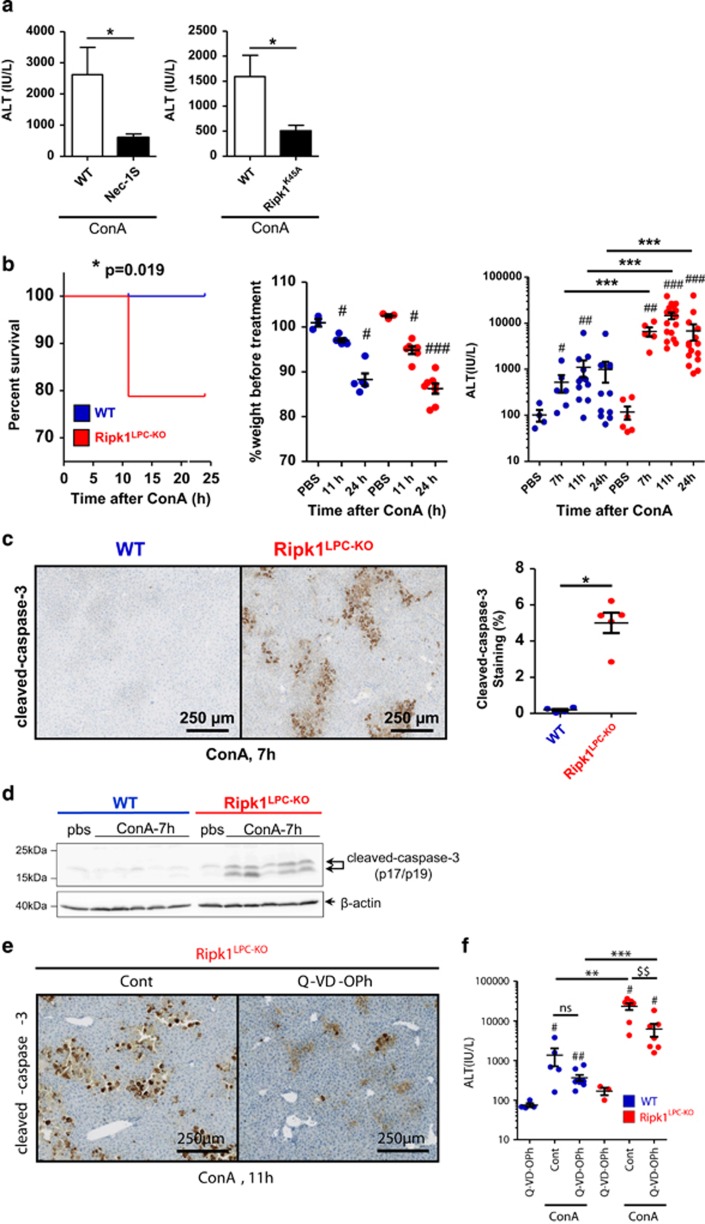
RIPK1 has pro-necroptotic and anti-apoptotic hepatocyte cell death functions during ConA-mediated hepatitis. (**a**) Levels of serum ALT 10 h after ConA injection, in WT mice pretreated or not by Nec-1 S (left panel), or in WT or *Ripk1*^K45A^ mice (right panel). (**b**) Survival curve: all WT mice (*n*=23) treated with ConA (12 mg/kg) survived and 7 *Ripk1*^LPC-KO^ mice on 33 died 12 h after ConA injection: kinetics of weight and serum ALT of WT and *Ripk1*^LPC-KO^ mice after PBS or ConA injection. (**c**) Pictures of liver tissue sections, analyzed by IHC for cleaved-caspase-3, issued from mice 7 h after ConA injection (upper panels). Signal quantification of cleaved-caspase-3 (lower panel). (**d**) Western blot analysis of cleaved-caspase-3 and *β*-actin in protein extracts issued from the livers of WT and *Ripk1*^LPC-KO^ mice, collected 7 h after ConA injection. Positions of molecular weight markers (kDa) and of studied proteins are respectively indicated on the left and right sides of the gel. (**e**) Cleaved-caspase-3 staining and (**f**) amounts of serum ALT in WT and *Ripk1*^LPC-KO^ mice, 11 h after ConA challenge with an eventual pre-treatment with a pan-caspase inhibitor (Q-VD-OPh) or only with its vehicle (Cont). For all graphs, each dot represent an individual and errors bars are expressed as means ±S.E.M. (*, ^#^, ^$^*P*<0.05; **, ^##^, ^$$^*P*<0.01; ***, ^###^, ^$$$^*P*<0.001; NS, nonsignificant)

Next, we evaluated the role of RIPK1 in liver parenchymal cells under challenged conditions. We, and others, have previously reported that ConA injection in mice induces TRAIL-mediated caspase-independent cell death of hepatocytes, which can be partially prevented by co-injection of the RIPK1 kinase inhibitor necrostatin-1 (Nec-1), suggestive of necroptosis induction.^20^ This inhibitor has, however, later been demonstrated not to be so specific for RIPK1.^25, 26^ We therefore re-evaluate the implication of RIPK1 enzymatic activity to ConA-induced hepatocyte death by inhibiting RIPK1 with Nec-1S, a RIPK1 kinase inhibitor more stable and specific than Nec-1, and by making use of the RIPK1 kinase dead (*Ripk1*^K45A^) mice. Importantly, both approaches confirmed that RIPK1 kinase activity drives liver injury induced by ConA, as shown by reduced levels of serum transaminase in Nec-1S pretreated mice and in *Ripk1*^K45A^ mice (Figure 2a).

In order to explore the pro-survival role of RIPK1 in the ConA-induced acute-hepatitis model, we used the *Ripk1*^LPC-KO^ mice that are fully deficient for both RIPK1 scaffold and kinase functions. Although injection of a low dose (12 mg/kg) of ConA in WT mice provoked a non-lethal moderate hepatitis, it elicited a fulminant hepatitis in *Ripk1*^LPC-KO^ mice leading to 21% lethality (Figure 2b, left). In both genotypes, the illness induced weight loss because of poor appetite (Figure 2b, middle) but liver damage were more pronounced in *Ripk1*^LPC-KO^ mice, as shown by wider necrotic areas (Supplementary Figure 1A), higher number of TUNEL-positive cells (Supplementary Figure 1A) and higher levels of serum transaminases (Figure 2b,Supplementary Figure 1B). Cell death in *Ripk1*^LPC-KO^ mice was apoptosis, as demonstrated by the important cleaved-caspase-3 staining detected by histological and western blot analysis (Figures 2c and d). This active caspase-3 staining was not observed in the WT littermate. To evaluate the importance of apoptosis in the fulminant ConA-induced hepatitis observed in *Ripk1*^LPC-KO^ mice, we treated mice 1 h before ConA injection with Q-VD-OPh hydrate (Q-VD-OPh), a pan-caspase inhibitor. Q-VD-OPh greatly decreased the number of cleaved-caspase-3-positive cells (Figure 2e) and significantly reduced hepatolysis, as shown by reduced injured areas in the liver (Supplementary Figure 1C) and the limited serum levels of transaminases (Figure 2f,Supplementary Figure 1D). In conclusion, our results indicated that RIPK1 kinase activity drives hepatocyte necroptosis following ConA injection but also serves as a scaffold protecting hepatocytes from massive apoptosis in the same model.

### RIPK1 limits inflammation in ConA-mediated hepatitis

**Figure 3:**
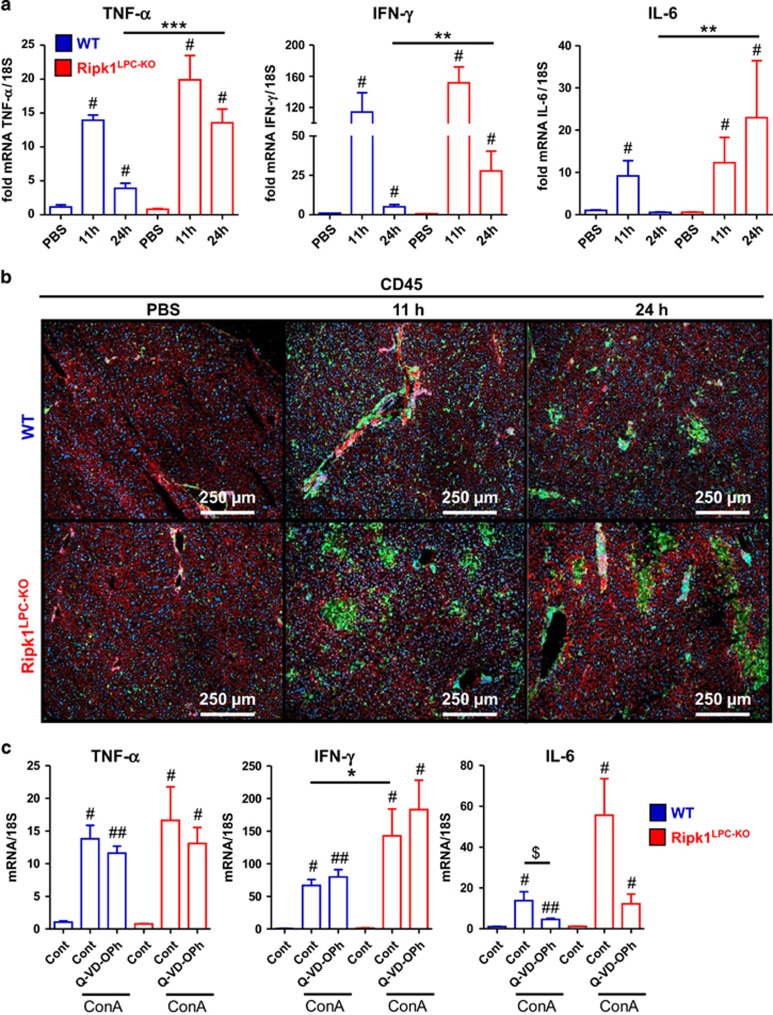
RIPK1 limits inflammation in ConA-mediated hepatitis. (**a**) Levels of hepatic TNF-*α*, IFN-*γ* and IL-6 transcripts in WT or *Ripk1*^LPC-KO^ mice 11 or 24 h after PBS or ConA injection. (**b**) CD45 immunofluorescence staining (green) on liver sections collected at the indicated times P.I. and issued from WT or *Ripk1*^LPC-KO^ mice challenged by PBS or ConA. Nucleus were stained by Hoechst (blue) and actin by fluorescent phalloidin (red). (**c**) Levels of hepatic TNF-*α*, IFN-*γ* and IL-6 transcripts in WT and *Ripk1*^LPC-KO^ mice, 11 h after ConA challenge with an eventual pre-treatment with a pan-caspase inhibitor (Q-VD-OPh) or only with its vehicle (Cont). For all graphs, errors bars are expressed as means ±S.E.M. (*, ^#^*P*<0.05; **, ^##^*P*<0.01; ***, ^###^*P*<0.001)

The lack of RIPK1 in liver parenchymal cells not only induced apoptotic cell death but also clearly exacerbated inflammation in ConA-challenged mice. Indeed, the induced mRNA levels of the inflammatory cytokines IFN-*γ*, TNF-*α* and IL-6 were comparable between the two genotypes 11 h after ConA injection, but significantly more abundant in the liver of *Ripk1*^LPC-KO^ mice 24 h after challenge (Figure 3a). Moreover, IFN-*γ* concentrations were significantly higher in the serum of *Ripk1*^LPC-KO^ mice at 11 and 24 h post-ConA injection (Supplementary Figure 2A). Although not statistically significant, a similar trend was also observed for TNF-*α* and IL-6 (Supplementary Figure 2A). In addition, immunolabeling of liver sections revealed higher hepatic recruitment of CD45- and CD11b-positive immune cells in *Ripk1*^LPC-KO^ individuals (Figure 3b,Supplementary Figure 2B). Sustained inflammation in *Ripk1*^LPC-KO^ mice was also demonstrated by the fact that hepatic mRNA levels of IL-6, a marker of liver injury involved in tissue repair, continued to increase at 24 h post-injection (P.I.) only in these animals (Figure 3a). In line with this, the number of CD69-positive lymphocytes remained more elevated in the spleen of *Ripk1*^LPC-KO^ mice at 24 h, reflecting prolonged activation of T lymphocytes promoted by the inflammatory microenvironment (Supplementary Figure 2C). Importantly, the occurrence of hepatocyte apoptosis in the liver of *Ripk1*^LPC-KO^ mice mostly contributed to the exacerbation of inflammation in these mice, and only to a limited extent to the establishment of the initial inflammatory microenvironment. Indeed, blocking apoptosis by Q-VD-OPh did not alter the early (11 h) induction of the hepatic TNF-*α* and IFN-*γ* transcripts (Figure 3c), nor the immune cell recruitment and activity (Supplementary Figures 3A and B). Nevertheless, Q-VD-OPh still prevented the 11-h induction of IL-6 mRNA in *Ripk1*^LPC-KO^ mice (Figure 3c), probably due to the fact that IL-6 has a key biological function in the liver regeneration and repair, and therefore lower hepatic damage induced less of IL-6 transcription. Together, these observations suggest that the protective effect of Q-VD-OPh on ConA-treated *Ripk1*^LPC-KO^ mice was due to the inhibition of caspase-mediated cell death and not to a major modification of the inflammatory microenvironment.

### The enhanced sensitivity of *Ripk1*^LPC-KO^ mice to ConA-induced hepatitis is mediated by TNF-*α*-promoted apoptosis

**Figure 4:**
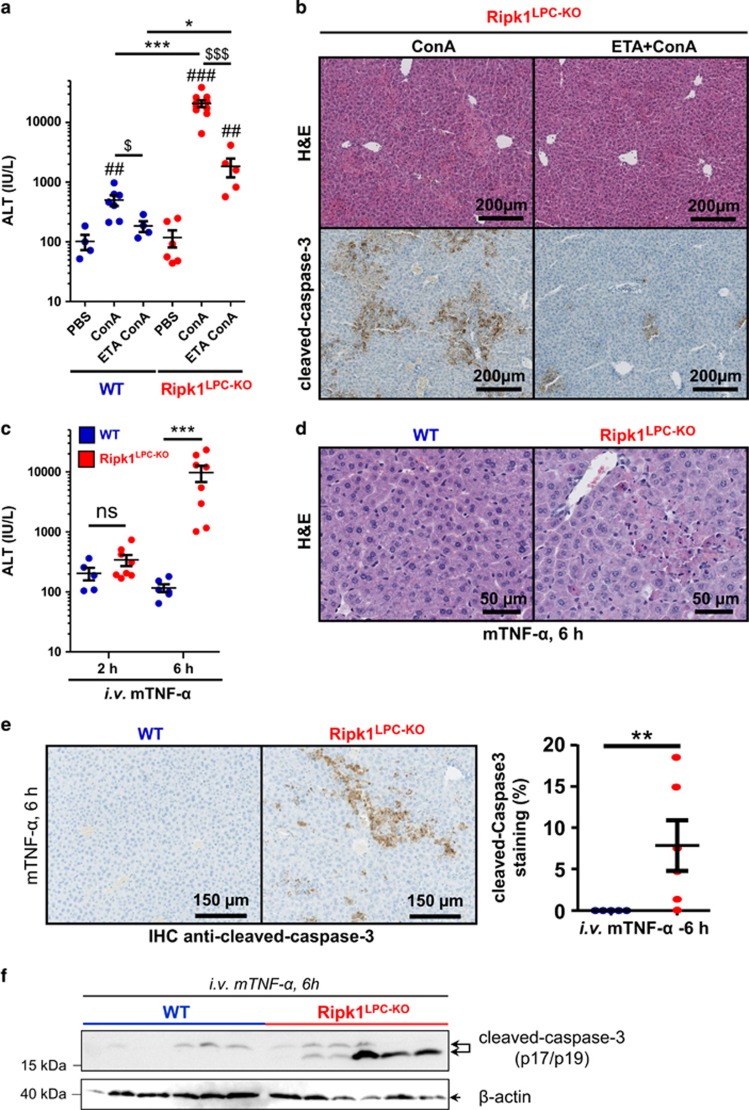
Sensitization of *Ripk1*^LPC-KO^ mice to ConA-induced hepatitis is triggered by TNF-*α*-mediated apoptosis. (**a**) Levels of serum ALT and (**b**) Pictures of liver tissue sections, stained by H&E (upper panel) or analyzed by IHC for cleaved-caspase-3 (lower panel), issued from mice in WT and *Ripk1*^LPC-KO^ mice, 11 h after ConA or PBS challenge with an eventual pre-treatment with ETA. (**c**) Levels of serum ALT in WT and *Ripk1*^LPC-KO^ mice, 2 and 6 h after mTNF-*α* injection. (**d**) Pictures of liver tissue sections stained by H&E and (**e**) analyzed by IHC for cleaved-caspase-3 (left panel) issued from WT and *Ripk1*^LPC-KO^ mice, 6 h after mTNF-*α* injection. Signal quantification of cleaved-caspase-3 (right panel). (**f**) Western blot analysis of cleaved-caspase-3 and *β*-actin in protein extracts issued from the liver of WT or *Ripk1*^LPC-KO^ mice, collected 6 h after mTNF-*α* injection. For all graphs, each dot represent an individual and errors bars are expressed as means ±S.E.M. (*, ^#^, ^$^*P*<0.05; **, ^##^, ^$$^*P*<0.01; ***, ^###^, ^$$$^*P*<0.001; NS, nonsignificant)

Our data demonstrated that, contrary to WT littermates, ConA injection resulted in a fulminant hepatitis in *Ripk1*^LPC-KO^ mice, in part caused by the sensitization of RIPK1-deficient liver parenchymal cells to apoptosis. Previous *in vitro* and *in vivo* studies have reported sensitization of RIPK1-deficient cells to TNF-mediated apoptosis.^27, 28^ Liver expression of TNF-*α*, and of its receptors TNFR1/2, is rapidly induced in the ConA-induced hepatitis model (Figures 3a and c,Supplementary Figures 2A and 4A). To investigate the role of TNF-*α* in the apoptosis of *Ripk1*^LPC-KO^ during ConA hepatitis, we neutralized TNF-*α* by injection of etanercept (ETA), a TNF-*α* decoy receptor. TNF-*α* blockade greatly protected *Ripk1*^LPC-KO^ mice from the exacerbated ConA-induced liver damage, as demonstrated by highly reduced serum levels of transaminases (Figure 4a,Supplementary Figure 4B), associated with a decrease of necrotic area and less apoptotic hepatocytes (Figure 4b). Of note, ETA also reduced the moderate liver damage in the similarly challenged WT littermates, indicative of a general role for TNF-*α* in the establishment of the ConA-induced hepatitis model (Figure 4a,Supplementary Figure 4B), as previously reported.^17, 29, 30^ To further assess the *in vivo* role of RIPK1 in the TNF-*α* signaling pathway in hepatocytes, soluble mTNF-*α* was injected in WT and *Ripk1*^LPC-KO^ littermates. Single mTNF-*α* injection had no effect on the liver of WT mice, but induced hepatolysis, already detected 6 h after injection, in *Ripk1*^LPC-KO^ mice. Hepatolysis was demonstrated by the analyses of serum transaminases (Figure 4c,Supplementary Figure 4C), liver infiltrate of immune cells (data not shown) and necrotic areas (Figure 4d,Supplementary Figure 4D). Hepatocytes died of apoptosis, as revealed by cleaved-caspase-3 staining (Figures 4e and f) and absence of phosphorylated MLKL immunodetection in the liver lysates (Supplementary Figure 5), a marker of necroptosis. Together, these experiments demonstrated that TNF-*α* contributes to ConA-induced hepatitis, and is responsible for the apoptosis and hepatitis severity observed in the *Ripk1*^LPC-KO^ mice.

### RIPK1 protects hepatocytes from ConA-mediated apoptosis independently of TNF-*α*-induced canonical NF-*κ*B activation

**Figure 5:**
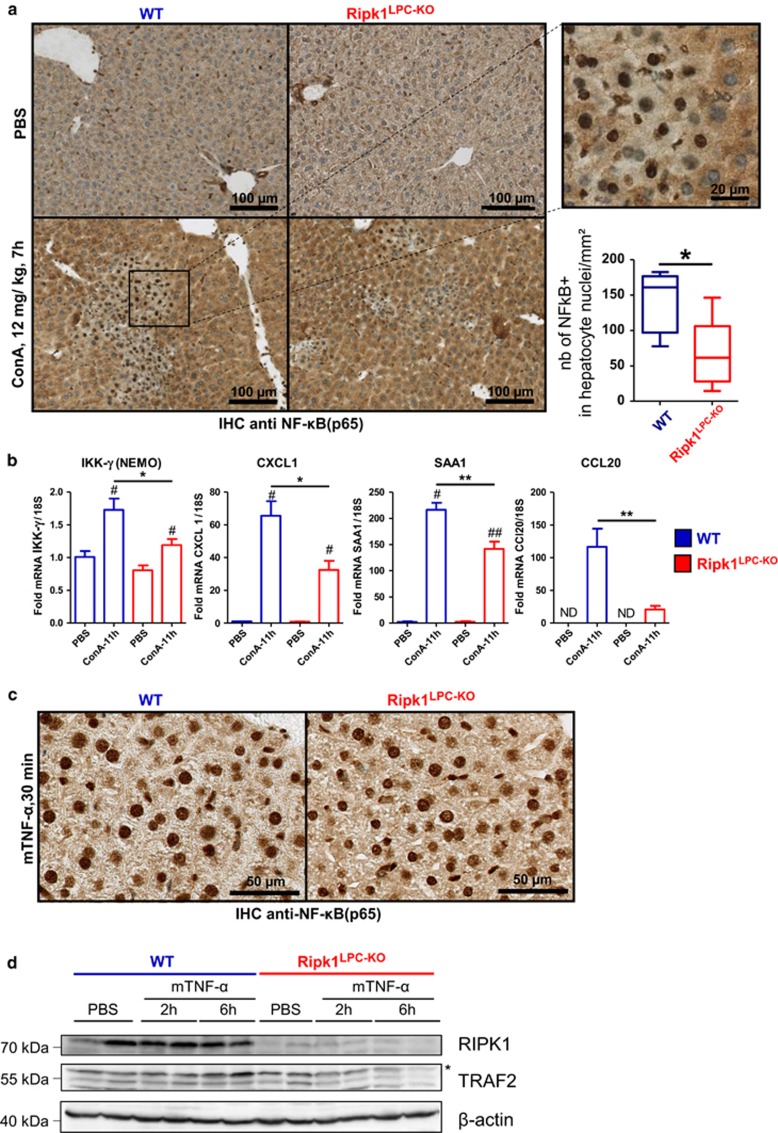
RIPK1 protects hepatocytes from ConA-mediated apoptosis independently of TNF-induced canonical NF-*κ*B activation. (**a**) Pictures of liver tissue sections, analyzed by IHC for NF-*κ*B-(p65), issued from WT or *Ripk1*^LPC-KO^ mice 7 h after ConA or PBS injection. Quantifications of hepatocyte nuclear signals are depicted in the bottom right inset. (**b**) Levels of hepatic IKK-*γ*/NEMO, CXCL1, SAA1 and CCL20 transcripts in WT and *Ripk1*^LPC-KO^ mice 11 h after ConA injection. (**c**) Pictures of liver tissue sections analyzed by IHC for NF-*κ*B-(p65) issued from mice 30 min after mTNF-*α* injection. (**d**) Western blot analysis of RIPK1, TRAF2 and *β*-actin in protein extracts issued from the liver of WT or *Ripk1*^LPC-KO^ mice, collected 2 h or 6 h after mTNF-*α* or PBS injection the star shows a nonspecific band. Errors bars are expressed as means ±S.E.M. (*P<0.05; **P<0.01; ***P<0.001)

In the TNFR1 signaling pathway, RIPK1 functions as a scaffold protein protecting cells from apoptosis by activating canonical NF-*κ*B-dependent and -independent responses.^10, 12, 13, 27^ Canonical NF-*κ*B activation is known to induce expression of various pro-survival molecules that prevent caspase activation. As a consequence, NF-*κ*B inhibition is often used to sensitize cells to TNF-*α*-induced apoptosis.^31^ The canonical NF-*κ*B-independent pro-survival function of RIPK1 is on the other hand far less understood, but suggested to rely on TNF receptor-associated factor 2 (TRAF2) stabilization, which prevents non-canonical NF-*κ*B activation and caspase-8 processing.^12, 13, 27^ We observed that the nuclear translocation of the p65 NF-*κ*B subunit was significantly reduced in *Ripk1*^LPC-KO^ hepatocytes 7 h following ConA injection, indicative of defective canonical NF-*κ*B activation (Figure 5a). Accordingly, induction of NF-*κ*B-target gene transcripts (IKK-*γ*/NEMO, CXCL1, SAA1 and CCL20) was repressed in the liver of *Ripk1*^LPC-KO^ mice (Figure 5b). The role of RIPK1 in ConA-induced canonical NF-*κ*B activation was independent of its kinase activity, as no difference was observed in the Nec-1S treated mice or the *Ripk1*^K45A^ mice (Supplementary Figure 6). Surprisingly, although TNF-*α* greatly contributed to apoptosis in RIPK1-deficient hepatocytes during ConA hepatitis (Figures 4a and b), no defect in p65 nuclear translocation or NF-*κ*B-dependent gene transcription (SAA1, CXCL1 and CCL20) was observed in the liver of *Ripk1*^LPC-KO^ mice 30 min after mTNF-*α* challenge (Figure 5c,Supplementary Figure 7). By contrast, TNF-*α* stimulation led to TRAF2 degradation only in *Ripk1*^LPC-KO^ animals, supporting a defect in the canonical NF-*κ*B-independent pro-survival pathway (Figure 5d).

**Figure 6:**
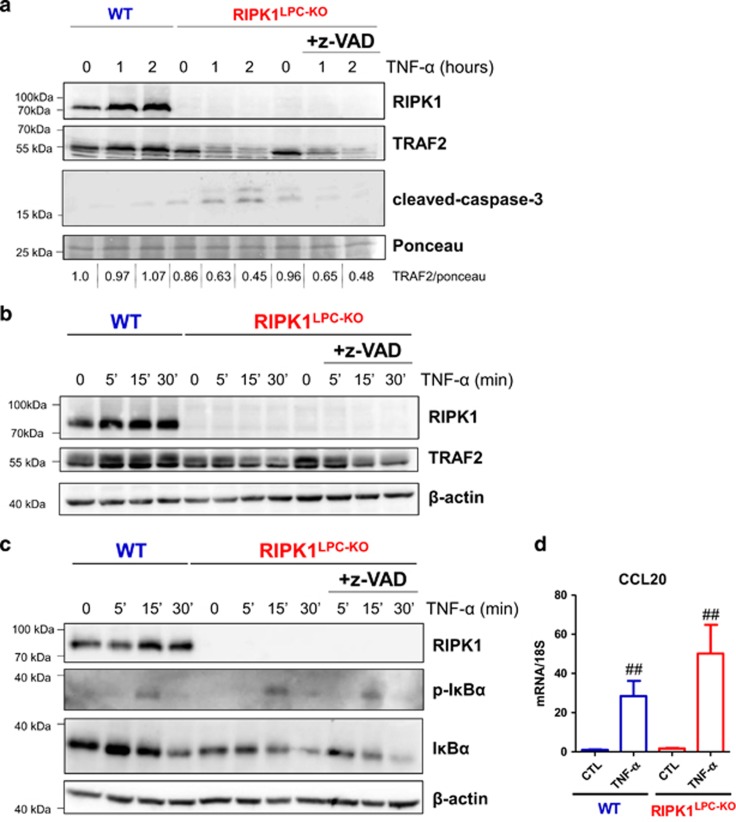
TNF-*α* induced primary hepatocyte death and TRAF2 destabilization in a NF-*κ*B-independent manner in absence of RIPK1. (**a**) Western blot analysis of RIPK1, TRAF2 and cleaved-caspase-3 in protein extracts of primary WT or RIPK1-deficient hepatocytes treated or not with TNF-*α* with or without z-VAD-fmk, during 1 h or 2 h. (**b** and **c**) Western blot analysis of RIPK1, TRAF2, P-I*κ*B*α* and *β*-actin in protein extracts of primary WT or RIPK1-deficient hepatocytes treated or not with TNF-*α* during 5, 15, 30 min. (**d**) Levels of CCL20 transcripts 2 h after or not (CTL) TNF-*α* stimulation in primary WT or RIPK1-deficient hepatocytes seeded with z-VAD-fmk before treatment. Errors bars are expressed as means ±S.D. (^#^*P*<0.05; ^##^*P*<0.01; ^###^*P*<0.001) and is based on the duplicate of primary hepatocyte cultures and triplicate for qPCR experiment

In contrast to primary hepatocytes isolated from WT mice, RIPK1-deficient hepatocytes died spontaneously after liver perfusion. Importantly, the death was prevented by seeding the cells in presence of ETA or with the pan-caspase inhibitor z-VAD-fmk (data not shown), demonstrating higher sensitivity of primary RIPK1-deficient hepatocytes to TNF-*α*-induced apoptosis. In line with this, TNF-*α* also only induced apoptosis in RIPK1-deficient primary hepatocytes when ETA was used only for seeding but removed before TNF-*α* stimulation, as revealed by active caspase-3 staining (Figure 6a). Consistent with our *in vivo* results, TRAF2 levels were also reduced in primary RIPK1-deficient hepatocytes stimulated with TNF-*α* (Figures 6a and b). The fact that z-VAD-fmk did not block TRAF2 degradation suggests that TRAF2 acts upstream of caspase activation (Figures 6a and b). Finally, we observed that RIPK1 deficiency did not affect canonical NF-*κ*B activation in primary hepatocytes stimulated with TNF-*α* in absence or presence of z-VAD-fmk, as shown by the kinetics of I*κ*B*α* phosphorylation and degradation and by the induction of CCL20, a NF-*κ*B-responsive gene (Figures 6c and d). Taken together, our results suggest that the protective role of RIPK1 against ConA-induced hepatocytes apoptosis may involve both TNF-independent canonical NF-*κ*B activation, as wellas TNF-dependent, but canonical NF-*κ*B-independent mechanisms.

## Discussion

TNF-*α* has a key role in the initiation of the inflammatory response during acute or chronic hepatitis caused by viral infections, steatosis, autoimmunity, alcohol or acetaminophen consumptions.^4, 32^ The production of TNF-*α* by liver Kupffer cells promotes expression of other cytokines and adhesion molecules that mediates the recruitment and the activation of immune cells involved in hepatocyte death.^33, 34^ A specific pro-inflammatory context is needed for the induction of hepatolysis by TNF-*α* as injection of TNF-*α* alone in mice is insufficient to induce hepatitis. Inhibition of transcription by d-galactosamine or inactivation of canonical NF-*κ*B signaling is often required to drive TNF-*α*-mediated hepatocyte apoptosis.^35, 36^ In the ConA hepatitis mouse model, which mimics human dysimmune hepatitis, immune cells activated by the lectin rapidly release TNF-*α* that is one of the first cytokines detected in the mouse serum.^29^ We found that neutralizing TNF-*α* with ETA reduced ConA-induced hepatitis, confirming previous studies indicating that genetic or chemical inactivation of TNF-*α* or TNFR1 protects ConA-treated animals from hepatitis.^17, 29, 30^ Together, these results underline the key role played by TNF-*α* in hepatitis induction. However, a recent publication reported that TNF-*α* does not directly induce hepatocyte death in ConA hepatitis in contrast to the d-GalN/LPS model. Indeed, specific deficiency for TNFR1 in myeloid derived cells (TNFR1^MDC-KO^), but not in liver parenchymal cells (TNFR1^LPC-KO^), protected mice from ConA hepatitis.^37^ These results indicate that TNF-*α* promotes inflammatory conditions to provoke liver damage in the ConA model, but that TNF-*α* is not a direct inductor of hepatocyte death. In the TNF-*α* signaling pathway, RIPK1 governs cell fate.^6, 38^ Our work highlights a dual role of RIPK1 during ConA hepatitis. First, and in accordance with previous studies,^20, 39^ we found that RIPK1 functions as a kinase inducing cell death. Indeed, inactivation of RIPK1 activity either by Nec-1S or genetic modification greatly protected ConA-challenged mice from hepatitis, demonstrating a key role of RIPK1 kinase activity in ConA-induced hepatocyte death. Depending on the cellular conditions, the enzymatic activity of RIPK1 has been reported capable of mediating apoptosis or necroptosis, a regulated form of necrosis with RIPK3 and MLKL as core components.^40, 41^ We found that WT mice with ConA-induced liver injuries exhibited only few caspase-3-positive hepatocytes, and that pan-caspase inhibition by Q-VD-OPh failed to protect these mice from ConA-induced hepatitis, supportive of necroptosis induction. Nevertheless, because a switch from apoptosis to necroptosis has been reported in hepatocytes upon caspases inactivation,^42, 43^ future works using hepatocytes-deficient RIPK3 or MLKL mice will help ruling out the occurrence of RIPK1 kinase-dependent apoptosis during ConA hepatitis. Next to its pro-death role, RIPK1 also appeared as a pro-survival scaffold for hepatocytes. In accordance with a recently published article, we found that RIPK1 is nonessential to maintain liver homeostasis under physiological condition.^44^ Nevertheless, mice with liver parenchymal cells lacking RIPK1 developed fulminant hepatitis associated with hepatocyte apoptosis when low doses of ConA or TNF-*α* were administrated. This phenotype partly contrasts with those observed in mice in which RIPK1 deficiency was limited to intestinal epithelial cells or to keratinocytes.^12, 13^ In these tissues, spontaneous apoptosis and inflammation arise, likely due to their direct interaction with commensal bacteria that trigger *de novo* TNF-*α* secretion. The role of TNF-*α* as a direct mediator of hepatocyte apoptosis in *Ripk1*^LPC-KO^ mice is highlighted by the reduction of ConA hepatitis by ETA or Q-VD-OPh, the induction of liver apoptosis by a single injection of TNF-*α* in *Ripk1*^LPC-KO^ mice, and the susceptibility of primary RIPK1-deficient hepatocytes to TNF-*α*-induced apoptosis. Activation of the canonical NF-*κ*B pathway may contribute to the protective role of RIPK1 in hepatocytes but would not be sufficient, and would occur independently of TNF-*α* signaling. Indeed, ConA-induced hepatitis in *Ripk1*^LPC-KO^ mice was associated with a partial defect in NF-*κ*B activation, as shown by reduced nuclear p65 labeling and by the lower expression of NF-*κ*B-dependent genes. However, hepatitis was also detected despite a widespread activation of the NF-*κ*B signaling pathway in hepatocytes of TNF-*α*-treated *Ripk1*^*LPC-KO*^ mice. This may be explained by the quick destabilization of TRAF2, observed *in vivo and in vitro* after TNF-*α* treatment. Accordingly, published data described different mechanisms by which RIPK1 may protect cells from TNF-induced death. In mouse embryonic fibroblasts lacking RIPK1, TNF-*α-*induced apoptosis is correlated to defective canonical NF-*κ*B activation and consequent c-FLIP dysregulation.^12^ Besides, RIPK1 would contribute to cell survival regardless of canonical NF-*κ*B, by its capacity to stabilize cIAPs and TRAF2 proteins, precluding non-canonical NF-*κ*B activation, c-FLIP destabilization and caspase recruitment.^12, 13, 27, 45^ Of note, the discrepancy between the defect, or not, in canonical NF-*κ*B activation following ConA or TNF-*α* injection could still be explained by the amount of circulating TNF-*α* provided in both systems, or RIPK1-dependent activation of NF-*κ*B by other stimulus than TNF. Anyway, our results still show TNF-*α*-induced apoptosis in RIPK1-deficient hepatocytes despite the ability of NF-*κ*B to translocate in nucleus, demonstrating a canonical NF-*κ*B-independent protective role of RIPK1 in these cells, as previously reported in other cells.^12, 13, 27, 45^

**Figure 7:**
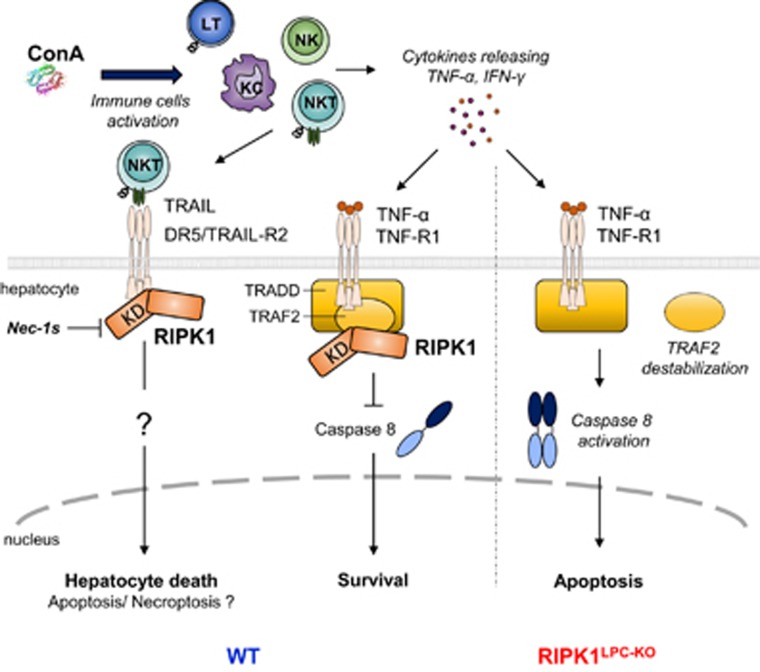
Role of RIPK1 in ConA hepatitis model. Schema of cell death pathway during ConA hepatitis in WT and *Ripk1*^LPC-KO^ mice. The lectin activates LT, KC, NK and NKT cells, which result in the release by these cells of cytokines, such as TNF-*α* and IFN-*γ*. Activation of NKT cells induces hepatocyte death mediated by TRAIL/DR5 signaling, and potentially also by TNFR1, which depends on RIPK1 kinase activity. TNF/TNFR1 binding also induces assembly of complex I by recruitment of TRADD, RIPK1 and TRAF2 preventing caspase activation and limiting cell death. RIPK1 deficiency induces TRAF2 destabilization upon stimulation and promotes formation of complex II by caspase-8 and apoptosis induction

Figure 7 proposes a schematic model of TNF-*α* signaling in hepatocytes of mice challenged with ConA. The lectin activates T lymphocytes (LT), Kupffer (KC), NK and NKT cells, which result in the release by these cells of cytokines, such as TNF-*α* and IFN-*γ*. Activation of NKT cells induces hepatocyte death mediated by TRAIL/DR5 signaling, and potentially also by TNFR1, which depends on RIPK1 kinase activity. TNF/TNFR1 binding also induces assembly of complex I by recruitment of TRADD, RIPK1 and TRAF2 preventing caspase activation and limiting cell death. RIPK1 deficiency induces TRAF2 destabilization upon stimulation and promotes formation of complex II, caspase-8 activation and apoptosis induction.

This work underlines the interest to focus studies on RIPK1 in other hepatitis models, and especially in acetaminophen (APAP) liver toxicity, where the role attributed to RIPK1 is not yet fully understood. Whereas most studies reveal a protective role of kinase inhibition by Nec-1^46, 47, 48^ or RIPK1 knockdown.^49^ Schneider *et al.^50^* recently suggest that RIPK1 deletion in hepatocytes has no effect in this hepatitis model. According to our demonstrated dual role of RIPK1 in hepatocytes, the kinase pro-death role of RIPK1 should be dissociated from his scaffolding pro-survival function in these studies by challenging RIPK1 kinase dead (*Ripk1*^K45A^) mice with APAP.

Moreover, our data could explain recent results obtained with of NEMO^LPC-KO^ mice, which presented spontaneous hepatocyte death.^44, 51, 52^ Surprisingly, apoptosis is strongly prevented by crossing NEMO^LPC-KO^ mice with RIPK1 kinase dead mice while poorly with *Ripk1*^LPC-KO^.^44^ As NEMO^LPC-KO^ mice presented elevated TNF-*α* in the liver compared with WT mice,^51^ additional RIPK1 deficiency could induce hepatocyte apoptosis in a TNF-*α*-dependent manner.

In conclusion, our results underline the protective role of RIPK1 in dysimmune hepatitis and the risks of potential defects in the RIPK1 scaffolding function that would sensitize hepatocyte to the death, risking to worsen hepatitis and even to the increase of HCC onset. Furthermore, our data pointed out the importance of studying the potential relationship between the genetic polymorphism of *Ripk1* gene and the predisposition to more severe liver damage in hepatic infectious or autoimmune diseases. Finally, our results support also that RIPK1 kinase activity is a pertinent therapeutic target to protect liver against excessive cell death in liver diseases.

## Materials and Methods

### Animals and treatment protocols

*Ripk1*^LPC-KO^ mice were generated by crossing *Ripk1*^fl/fl^ strain^11^ with Alfp-Cre transgenic mice having a Cre recombinase driven by the serum albumin gene promoter completed with albumin and *α*-fetoprotein enhancers.^21^ RIPK1 kinase dead knock-in (*Ripk1*^K45A^) mice have been already described.^10^ In all experiments, genetically modified mice were systematically compared with their littermates. Animals were housed in individually ventilated cages at the VIB Inflammation Research Center in conventional animal facilities. All experiments on mice were conducted according to Institutional, National and European animal regulations. *In vivo* protocols were approved by the ethics committee of Ghent University. Homogeneous groups of male and female mice at 7–13 weeks of age were used for each experiment. ConA (C2010 Sigma-Aldrich, St. Louis, MO, USA) diluted at 3 mg/ml in PBS supplemented with MnCl_2_ 0.31 mM and CaCl_2_ 0.75 mM, was administered by intravenous (i.v.) injection at a dose of 10 or 12 mg/kg body weight. Q-VD-OPh hydrate (Sigma-Aldrich, SML0063) was injected in mice via the intraperitoneal (i.p.) route (20 mg/kg and 10 *μ*l/g of body weight) 1 h before ConA injection. Nec-1 s (Biovision Inc., Milpitas, CA, USA, #2263) was administered by i.v. injection (6.25 mg/kg and 4 *μ*l/g body weight) 15 min before the ConA challenge. An i.p. injection was used to deliver ETA (Enbrel, Pfizer, New-York, NY, USA) to mice (10 mg/kg and 10 *μ*l/g body weight) 1 h before ConA injection. Mice were given a single i.v. injection of murine TNF-*α* (mTNF-*α*, Peprotech, Rocky Hill, NJ, USA, 315-01A, 40 *μ*g/ml, at a dose of 10 *μ*g/kg body weight). The control mice received similar volumes of vehicle in each treatment group. Times of killing are indicated for each experiment.

### RNA analysis

Total RNA was extracted, from mice livers tissues using TRIzol reagent (Thermo Fisher Scientific, Waltham, MA, USA) and from primary hepatocytes by using the RNeasy mini kit (Qiagen, #74106). First-strand cDNA was synthesized using the SuperScript^TM^ II Reverse Transcriptase (Applied Biosystems, Foster City, CA, USA). Real-time quantitative PCR was performed using the fluorescent dye SYBR Green with the double-strand specific SYBR^®^ Green system (Applied Biosystems) and the ABI 7000 Prism sequence detector (Applied Biosystems) or the CFX384 Touch™ Real-Time PCR Detection System (Bio-Rad). cDNA was used as template for amplification with specific primer pairs (Table 1). Each measurement was performed in triplicate. The relative gene expression was normalized against the 18S gene expression. The control mice in each treatment group served as a reference for messenger RNA (mRNA) expression (control mRNA level was arbitrarily set at 1).

### Histopathological and biochemical studies

Fragments of mouse livers were fixed in 4% paraformaldehyde and embedded in paraffin for IHC and hematoxylin and eosin (H&E), or frozen in isopentane cooled with liquid nitrogen for immunofluorescence studies. For histopathology, H&E staining of liver tissues was carried out to investigate liver injury. Serum ALT and AST transaminases was measured according to the IFCC primary reference procedures using Olympus AU2700 Autoanalyser^®^ (Olympus Optical, Tokyo, Japan).

### Immunolocalization in liver tissues

For immunolocalization of cleaved-caspase-3, RIPK1, NF-*κ*B-p65 and Ki67 in liver tissues, paraffin-embedded mouse liver sections (4–5 *μ*m) were dried 1 h at 58 °C, followed by antigen retrieval and incubated with primary antibody (Cell Signaling Technology, Danvers, MA, USA, 9661S; 3493; Santa Cruz Biotechnology, Dallas, TX, USA, sc-372, respectively) in a Ventana automated machine (Ventana Medical Systems, USA). Revelation of primary antibody was carried out using horseradish peroxidase (HRP)-conjugated secondary antibody (Dako, Glostrup, Denmark) and DAB substrate kit (Ventana Medical Systems, Tucson, AZ, USA, #760-124). Slides were then counterstained with hematoxylin. TUNEL analysis was performed on mouse liver sections incubated after antigen retrieval with a mix composed of terminal transferase (Roche, Mannheim, Germany, 3333566 011) and digoxigenin-11-UTP (Roche, #1558706) followed by biotinylated anti-digoxigenin (Sigma, #B7405) and counterstained with hematoxylin.

For immunolocalization of CD11b and CD45 in liver tissues, cryosections of mouse liver tissues (8 *μ*m) were successively fixed with paraformaldehyde 4%, treated with NH_4_Cl 0.1 M, blocked with 2–4% BSA and incubated with primary antibody (BD Pharmingen, San Jose, CA, USA, #552850 and #553079; respectively) at 4 °C overnight. Revelation of primary antibody was carried out using DyLight 649-conjugated donkey anti-rat IgG secondary antibody (Jackson ImmunoResearch Laboratories, Baltimore, PA, USA). Nuclei were stained with Hoechst (Invitrogen, H3570) and F-actin with Phalloïdine FluoProbes-547H (Interchim, Montluçon, France,BZ9620). Image analysis and merged were performed with SpotAdvanced software (Diagnostic Instruments, Sterling Heights, MI, USA). Quantification of cleaved-caspase-3 positive-signal was performed with image analysis software (NIS-Element AR analysis software, Nikon, Tokyo, Japan) and measured to cover an area of 4.3–6 mm^2^. The quantification hepatocytes with nuclear NF-*κ*B, labeled nuclei were counted on 3–4 independent fields to cover an area of 3.54–4.72 mm^2^.Pictures presented in the figures are issued from animals with ALT or quantified staining values close to the average obtained for the membership group.

### Protein extraction and western blotting

Primary hepatocytes and mouse liver specimens were lysed in RIPA buffer (50 mM Tris-HCl pH 7.4; 1% Triton X-100; 25 mM HEPES; 150 mM NaCl; 0,2% SDS; 5 mM MgCl_2_; 1 mM Na_3_VO_4_; 1 mM NaF and proteases inhibitors (Roche, #04 693 132 001)), and liver were crushed with UltraTurax. After 40 min in ice, samples were centrifuged at 13 000 *g*. Proteins from supernatant were assayed with the Bradford method (Bio-Rad). Proteins were separated by SDS-PAGE and transferred onto nitrocellulose membrane. Membranes were blocked with non-fat milk or BSA 3–5% in TBS (20 mM Tris, 137 mM NaCl) during 1–2 h and incubated overnight with primary antibody anti-cleaved-caspase-3, anti-RIPK1 (Cell Signaling) anti-actin (Sigma A3854), anti-TRAF2 (Santa Cruz Biotechnology, sc-876), anti-phospho-I*κ*B*α* (Cell Signaling, #2859) or anti-I*κ*B*α* (Santa Cruz Biotechnology, sc-371) at 4 °C, and then with secondary goat anti-rabbit immunoglobulins/HRP (Dako, P0448). Protein–antibody complexes were revealed by enhanced chemiluminescence (Millipore, Billerica, MA, USA) and ImageQuant LAS-4000 mini imager analysis (GE Healthcare, Chicago, IL, USA).

### Primary hepatocyte isolation and culture

Hepatocytes were isolated from adult C57BL/6 WT and *Ripk1*^LPC-KO^ mice by the retrograde perfusion approach.^23^ Mice were previously anesthetized by i.p injection of a ketamine (Imalgene^TM^, Merial Lyon, France, supplier) and xylazine (Rompun^TM^, Bayer, Leverkusan, Germany, supplier) cocktail (60 and 10 mg/kg of live body weight, respectively) diluted in PBS. After laparotomy, the liver, perfused through *inferior vena cava*, was first washed with solution I (8 g/l NaCl, 0.2 g/l KCl, 0.1 g/l Na_2_HPO_4_. 12 H_2_O and 2.38 g/l Hepes, pH 7.6) at a 5.5 ml/min flow rate for 8–10 min. Then, the perfusion solution I was supplemented with 5 mM CaCl_2_.2H_2_O and 0.01% collagenases (Liberase^TM^ TM Research Grade, Roche) for 5–7 min. Hepatocytes were harvested after three centrifugations at 90 g for 1 min and an additional purification of viable cells on a percoll cushion. Cell suspensions, with over 90% viability, were seeded at a density of 0.1 × 10^6^ cells/cm^2^ in 24-well plates, previously coated with collagen type I (BD Biosciences, Le Pont de Claix, France), in Williams' E medium supplemented with 10% (vol/vol) fetal calf serum, 2 mM glutamine, 10 IU/ml penicillin, 10 *μ*g/ml streptomycin and 5 *μ*g/ml insulin. Seeding medium was removed after a 4-h period and replaced by a similar supplemented Williams' E medium, without fetal calf serum but with 1 mg/ml bovine serum albumin, but with 1 *μ*g/ml of ETA and optionally with 10 *μ*M of z-VAD-fmk. After two or three washes, cells were stimulated with 10 ng/ml of mTNF-*α* (Peprotech, 315-01A), and used for RNA or protein extraction. Experimental protocol was conducted in compliance with French laws and the institution's guidelines for animal welfare (agreement of M Samson *#* A3523840).

### Serum cytokine immunoassays by flow cytometry

Murine TNF-*α*, IFN-*γ* and IL-6 cytokines were quantified by bead-based immunoassays according to the manufacturer protocol, using a filter plate and a vacuum filtration system for washing steps (BioLegend's LEGENDPLEX, multi-analyte flow assay kit, San Diego, CA, USA). Samples were analyzed on FC500 cytometer (Beckman Coulter, Brea, CA, USA).

### Immune cells analysis by flow cytometry

Immune cells were prepared from spleen or liver crushed on a 70 *μ*m filter. Liver immune cells were isolated after sedimentation and cell fractionation on a 35% Percoll layer. For each organ, red blood cells were lysed with the ammonium-chloride-potassium (ACK) buffer. To exclude dead cells from analysis, cell suspensions were labeled for 30 min with LIVE/DEAD fixable yellow stain (Life Technologies, Carlsbad, CA, USA, L34959). Cells were also pre-incubated with an anti-CD16/32 antibody (BD Pharmingen) to block nonspecific binding, before the incubation with the appropriate fluorochrome-conjugated antibodies (BD Pharmingen, eBioscience, San Diego, CA, USA): anti-CD3-FITC (clone 17A2), anti-TCR*β*-V450 (clone H57-597), anti-CD69-PE (clone H1.2F3), anti-CD19-APC (clone 1D3), anti-NK1.1-PerCP-Cy-5.5 (clone PK136), anti-CD4-PE-Cy7 (clone RM4-5), anti-CD8-APC-Cy7 (clone 53-6.7), anti-GR1-eFluor450 (clone FB6-8C5) or anti-CD11b-PE-Cy7 (clone M1/70). The stained cells were analyzed on FACSAria™ II flow cytometer using BD FACSDiva software (BD Bioscience), and data were analyzed with the CXP software (Beckman Coulter). Doublet and dead cells were excluded, respectively, on the basis of forward/side scatter and with the LIVE/DEAD labeling. The used immuno-phenotyping was as followed: Lymphocyte B (LyB): CD19+/CD3- cells; Lymphocyte T (LyT): CD3+/TCRV*β*+/NK1.1-; NKT cells: CD3+/ TCRV*β*+/NK1.1+ NK cells: CD3-/NK1.1+ granulocytes: GR1+CD11b+. Lymphoid activation was studied by analyzing the expression of CD69. To calculate percentage of each immune cell population, we considered as 100% of immune cells the sum of events of each immune cell population analyzed (sum of LyT, LyB, NK cells, NKT cells and granulocytes).

### Statistical analysis

Data were expressed as means ±S.E.M. for all mice treated similarly. Kruskal–Wallis one-way analysis of variance (ANOVA) was performed, and mean differences between experimental groups were assessed using the non-parametric Mann–Whitney *U*-test with the GraphPad Prism5 software (GraphPad, La Jolla, CA, USA). The significance is shown as follows: *P<0.05, **P<0.01, ***P<0.001, WT versus RIPK1 mice. ^#^*P*<0.05, ^##^*P*<0.01, ^###^*P*<0.001, mice treated with ConA, or TNF-*α* versus mice treated with PBS. ^$^*P*<0.05, ^$$^*P*<0.01, ^$$$^*P*<0.001 mice treated with ConA, or TNF-*α* at an indicated time versus mice treated with ConA, or TNF-*α* at another time in same genotype.

## Figures and Tables

**Table 1:** Sequence of primers used for qPCR

**Gene**	**Forward**	**Reverse**
Mouse 18S	5′-CGCCGCTAGAGGTGAAATTC-3′	5′-TTGGCAAATGCTTTCGCTC-3′
Mouse TNF-*α*	5′-TAGCTCCCAGAAAAGCAAGC-3′	5′-TTTTCTGGAGGGAGATGTGG-3′
Mouse IL-6	5′-CCGGAGAGGAGACTTCACAG-3′	5′-CAGAATTGCCATTGCACAAC-3′
Mouse IFN-*γ*	5′-AGGTCAACAACCCACAGGTC-3′	5′-ATCAGCAGCGACTCCTTTTC-3′
Mouse-CXCL1	5′-CGCCTATCGCCAATGAGC-3′	5′-GAACCAAGGGAGCTTCAGG-3′
Mouse TNFR1	5′-CAGAACACCGTGTGTAACTGC-3′	5′-GCAAGCGGAGGAGGTAGG-3′
Mouse TNFR2	5′-CGCTGGTCTTCGAACTGC-3′	5′-CAGGAGGACACTTAGCACAGC-3′
SAA1	5′-TGTTCACGAGGCTTTCCAAG-3′	5′-GTCCTCTGCCGAAGAATTCC-3′
CCL20	5′-TCTGCTCTTCCTTGCTTTGG-3′	5′-TCACCCAGTTCTGCTTTGG-3′
Mouse-IKK-*γ*/NEMO	5′-GGTGGAGAGACTGAGCTTGG-3′	5′-CCTCTAAAGCTTGCCGATCC-3′

## Abbreviations

AST: aspartate amino-transferase
ALT: alanine amino-transferase
ConA: concanavalin A
DR: death receptor
ETA: etanercept
HCC: hepatocellular carcinoma
H&E: hematoxylin and eosin coloration
i.v: intravenous
i.p: intraperitoneal
NASH: nonalcoholic fatty liver hepatitis
Nec-1: necrostatin-1
P.I: post-injection
RIPK: receptor interacting protein kinase
Q-VD-OPh: quinoline-Val-Asp-CH_2_-Ph
TNF: tumor necrosis factor
TRAF2: TNF receptor-associated factor 2
TRAIL: TNF-related apoptosis-inducing ligand
