# Supplementary material for: RIPK1 protects from TNF-α-mediated liver damage during hepatitis
Source: Cell Death Dis. 2016 Nov 10;7(11):e2462–. doi: 10.1038/cddis.2016.362 (PMC5260888; doi:10.1038/cddis.2016.362)
Supplement: Supplementary Information [file cddis2016362x1.doc]

**Figure 7: Role of RIPK1 in ConA hepatitis model**.

Schema of cell death pathway during ConA hepatitis in WT and *Ripk1*^LPC-KO^ mice. The lectin activates T-lymphocytes (LT), Kupffer (KC), Natural killer (NK), and Natural killer T (NKT) cells, which result in the release by these cells of cytokines, such as TNF-α and IFN-γ. Activation of NKT cells induces hepatocyte death mediated by TRAIL/DR5 signaling, and potentially also by TNFR1, which depends on RIPK1 kinase activity. TNF/TNFR1 binding also induces assembly of complex I by recruitment of TRADD, RIPK1 and TRAF2 preventing caspase activation and limiting cell death. RIPK1 deficiency induces TRAF2 destabilization upon stimulation and promotes formation of complex II by caspase-8 and apoptosis induction.

**Supplementary Figure 1.**

(A) Pictures of liver tissue sections, stained by H&E (upper and middle panels) or analyzed by TUNEL immunostaining (lower panel) issued from mice after ConA injection. Dotted white lines surround the necrotic areas and black arrows show labeled nuclei in TUNEL. (B) Kinetics of serum AST of WT and Ripk1LPC-KO mice after PBS or ConA injection. (C) Levels of serum AST in animals 11 h after ConA injection. Mice were pre-treated 1 h before ConA with Q-VD-OPh or vehicule (Cont). (D) Pictures of liver tissue sections, stained by H&E issued from Ripk1LPC-KO mice pre-treated 1 h before ConA with Q-VD-OPh or not Cont. Each dot represent an individual and errors bars are expressed as means +/- SEM. (*, #, $ p<0.05; **, ##, $$ p<0.01; ***, ###, $$$ p<0.001, ns: non-significant).

**Supplementary Figure 2.**

(A) Kinetics of cytokines (TNF-α, IFN-γ and IL-6) levels measured in sera of WT and *Ripk1*^LPC-KO^ mice challenged or not (PBS) by ConA. Assays were carried out at 7, 11 and 24 h post-injection. Errors bars are expressed as means +/- SEM. (B) CD11b (green) and F-actin (red) immunofluorescence staining on liver sections collected at the indicated times after ConA injection and issued from WT or *Ripk1*^LPC-KO^ mice.(C) CD69 expression analysis by FACS on CD3-/CD19+ B-lymphocyte splenocytes, issued from WT and *Ripk1*^LPC-KO^ mice challenged by PBS or ConA (right part). Percentage of CD-69 positive-B-lymphocytes 11 h or 24 h after ConA treatment (left part). (*, #p<0.05; **, ## p<0.01; ***, ### p<0.001).

**Supplementary Figure 3.**

(A) Gating strategy of C57BL/6 WT liver immune cells analysis, 12 h after ConA treated mice +/- Q-VD-OPh. After exclusion of doublet and dead cells, lymphocyte population were analyzed on events with FSClow/SSClow, and granulocyte population on all cells. B lymphocytes (LyB) corresponding to CD19+/CD3- cells, T lymphocytes (LyT): CD3+/NK1.1-, Natural Killer-T (NKT): CD3+/NK1.1+, Natural Killer (NK): CD3-/NK1.1+ and granulocytes as GR1+/CD11b+. The graph represents percent of each liver immune cells population. (B) CD69 expression analysis by FACS on LyB liver immune cells of mice challenged with PBS or ConA and pre-treated with Q-VD-OPh or not (Cont). Errors bars are expressed as means +/- SEM (*, $, #p<0.05; **, $$, ## p<0.01; ***, $$$, ### p<0.001; ns: non-significant).

Comment: % of liver immune cells treated with Q-VD-OPh alone have the same profil as Cont treated mice. The same applies to the mice treated with Cont+ConA and Q-VD-OPh+ConA. In both cases, LyB, NK and granulocytes are recruit in liver and NK-T fall to around 2-3 %. All lymphocytes express CD69 in this both conditions.

**Supplementary Figure 4.**

(A) Levels of hepatic TNF-α, TNFR1, TNFR2 transcripts in WT or *Ripk1*^LPC-KO^ mice 7 h after ConA injection. PBS- and ConA-treated groups respectively consisted of 3 or 5 to 7 individuals. (B) Levels of serum AST in animals 11 h after ConA injection. Mice were pre-treaded 1 h before ConA with Etanercept (ETA) or control (PBS). (C) Levels of serum AST in animals 2 and 6 h after mTNF-α injection. (D) Pictures of liver tissue sections, stained by H&E issued from WT and *Ripk1*^LPC-KO^ mice 2 h after *i.v.* mTNF-α injection, black arrows show apoptotic hepatocytes. Errors bars are expressed as means +/- SEM (*, $, #p<0.05; **, $$, ## p<0.01; ***, $$$, ### p<0.001; ns: non-significant).

**Supplementary Figure 5.**

Western-blot analysis of RIPK1, P-MLKL and β-actin in protein extracts issued from the livers of WT and *Ripk1*^LPC-KO^ mice, 11 hours after PBS or ConA injection with or without pretreatment with the pan caspase inhibitor Q-VD-Oph (QVD). CTL corresponds to protein extract from L929 cells treated 8 hours with 125 nM of the smac mimetic Birinapant, 10 µM of zVAD-fmk and 100 ng/mL of TNF-α.

**Supplementary Figure 6.**

Levels of hepatic IKK-γ/NEMO transcript in in WT mice pre-treated or not by Nec-1S or in WT or *Ripk1*^K45A^ mice presented. Errors bars are expressed as means +/- SEM.

**Supplementary Figure 7.**

Levels of hepatic SAA1, CXCL1 and CCL20 transcripts in WT or *Ripk1^LPC-KO^* mice 2 h or 6 h after mTNF-α injection. Errors bars are expressed as means +/- SEM (*, $, #p<0.05; **, $$, ## p<0.01; ***, $$$, ### p<0.001; ns: non-significant).

**References**

1. Friedman SL. Liver fibrosis -- from bench to bedside. *J Hepatol* 2003, **38 Suppl 1:** S38-53.

2. Luedde T, Kaplowitz N, Schwabe RF. Cell death and cell death responses in liver disease: mechanisms and clinical relevance. *Gastroenterology* 2014, **147**(4)**:** 765-783 e764.

3. Brenner C, Galluzzi L, Kepp O, Kroemer G. Decoding cell death signals in liver inflammation. *Journal of Hepatology* 2013, **59**(3)**:** 583-594.

4. Arshad MI, Piquet-Pellorce C, L'Helgoualc'h A, Rauch M, Patrat-Delon S, Ezan F*, et al.* TRAIL but not FasL and TNFalpha, regulates IL-33 expression in murine hepatocytes during acute hepatitis. *Hepatology* 2012, **56**(6)**:** 2353-2362.

5. Pasparakis M, Vandenabeele P. Necroptosis and its role in inflammation. *Nature* 2015, **517**(7534)**:** 311-320.

6. Muto Y, Nouri-Aria KT, Meager A, Alexander GJ, Eddleston AL, Williams R. Enhanced tumour necrosis factor and interleukin-1 in fulminant hepatic failure. *Lancet* 1988, **2**(8602)**:** 72-74.

7. Bird GL, Sheron N, Goka AK, Alexander GJ, Williams RS. Increased plasma tumor necrosis factor in severe alcoholic hepatitis. *Ann Intern Med* 1990, **112**(12)**:** 917-920.

8. Morioka S, Broglie P, Omori E, Ikeda Y, Takaesu G, Matsumoto K*, et al.* TAK1 kinase switches cell fate from apoptosis to necrosis following TNF stimulation. *J Cell Biol* 2014, **204**(4)**:** 607-623.

9. Kelliher MA, Grimm S, Ishida Y, Kuo F, Stanger BZ, Leder P. The death domain kinase RIP mediates the TNF-induced NF-kappaB signal. *Immunity* 1998, **8**(3)**:** 297-303.

10. Berger SB, Kasparcova V, Hoffman S, Swift B, Dare L, Schaeffer M*, et al.* Cutting Edge: RIP1 Kinase Activity Is Dispensable for Normal Development but Is a Key Regulator of Inflammation in SHARPIN-Deficient Mice. *The Journal of Immunology* 2014, **192**(12)**:** 5476-5480.

11. Takahashi N, Vereecke L, Bertrand MJ, Duprez L, Berger SB, Divert T*, et al.* RIPK1 ensures intestinal homeostasis by protecting the epithelium against apoptosis. *Nature* 2014, **513**(7516)**:** 95-99.

12. Dannappel M, Vlantis K, Kumari S, Polykratis A, Kim C, Wachsmuth L*, et al.* RIPK1 maintains epithelial homeostasis by inhibiting apoptosis and necroptosis. *Nature* 2014, **513**(7516)**:** 90-94.

13. Roderick JE, Hermance N, Zelic M, Simmons MJ, Polykratis A, Pasparakis M*, et al.* Hematopoietic RIPK1 deficiency results in bone marrow failure caused by apoptosis and RIPK3-mediated necroptosis. *Proceedings of the National Academy of Sciences of the United States of America* 2014, **111**(40)**:** 14436-14441.

14. Tiegs G, Hentschel J, Wendel A. A T cell-dependent experimental liver injury in mice inducible by concanavalin A. *The Journal of Clinical Investigation* 1992, **90**(1)**:** 196-203.

15. Arshad MI, Rauch M, L'Helgoualc'h A, Julia V, Leite-de-Moraes MC, Lucas-Clerc C*, et al.* NKT cells are required to induce high IL-33 expression in hepatocytes during ConA-induced acute hepatitis. *Eur J Immunol* 2011, **41**(8)**:** 2341-2348.

16. Watanabe Y, Morita M, Akaike T. Concanavalin A induces perforin-mediated but not Fas-mediated hepatic injury. *Hepatology* 1996, **24**(3)**:** 702-710.

17. Kusters S, Tiegs G, Alexopoulou L, Pasparakis M, Douni E, Kunstle G*, et al.* In vivo evidence for a functional role of both tumor necrosis factor (TNF) receptors and transmembrane TNF in experimental hepatitis. *Eur J Immunol* 1997, **27**(11)**:** 2870-2875.

18. Tagawa Y, Kakuta S, Iwakura Y. Involvement of Fas/Fas ligand system-mediated apoptosis in the development of concanavalin A-induced hepatitis. *Eur J Immunol* 1998, **28**(12)**:** 4105-4113.

19. Jouan-Lanhouet S, Arshad MI, Piquet-Pellorce C, Martin-Chouly C, Le Moigne-Muller G, Van Herreweghe F*, et al.* TRAIL induces necroptosis involving RIPK1/RIPK3-dependent PARP-1 activation. *Cell Death Differ* 2012, **19**(12)**:** 2003-2014.

20. Zhou Y, Dai W, Lin C, Wang F, He L, Shen M*, et al.* Protective Effects of Necrostatin-1 against Concanavalin A-Induced Acute Hepatic Injury in Mice. *Mediators of Inflammation* 2013, **2013:** 1-15.

21. Arshad MI, Piquet-Pellorce C, Filliol A, L'Helgoualc'h A, Lucas-Clerc C, Jouan-Lanhouet S*, et al.* The chemical inhibitors of cellular death, PJ34 and Necrostatin-1, down-regulate IL-33 expression in liver. *J Mol Med (Berl)* 2015, **93**(8)**:** 867-878.

22. Kellendonk C, Opherk C, Anlag K, Schütz G, Tronche F. Hepatocyte-specific expression of Cre recombinase. *Genesis (New York, NY: 2000)* 2000, **26**(2)**:** 151-153.

23. Houseman L, Edwards M, Phillips IR, Shephard EA. Isolation and Culture of Mouse Hepatocytes: Gender-Specific Gene Expression Responses to Chemical Treatments. *Methods Mol Biol* 2015, **1250:** 3-12.

24. Sato M, Tanigawa M. Production of CETD transgenic mouse line allowing ablation of any type of specific cell population. *Mol Reprod Dev* 2005, **72**(1)**:** 54-67.

25. Degterev A, Maki JL, Yuan J. Activity and specificity of necrostatin-1, small-molecule inhibitor of RIP1 kinase. *Cell Death Differ* 2013, **20**(2)**:** 366.

26. Takahashi N, Duprez L, Grootjans S, Cauwels A, Nerinckx W, DuHadaway JB*, et al.* Necrostatin-1 analogues: critical issues on the specificity, activity and in vivo use in experimental disease models. *Cell Death Dis* 2012, **3:** e437.

27. Gentle IE, Wong WW, Evans JM, Bankovacki A, Cook WD, Khan NR*, et al.* In TNF-stimulated cells, RIPK1 promotes cell survival by stabilizing TRAF2 and cIAP1, which limits induction of non-canonical NF-kappaB and activation of caspase-8. *J Biol Chem* 2011, **286**(15)**:** 13282-13291.

28. Estornes Y, Aguileta MA, Dubuisson C, De Keyser J, Goossens V, Kersse K*, et al.* RIPK1 promotes death receptor-independent caspase-8-mediated apoptosis under unresolved ER stress conditions. *Cell Death Dis* 2014, **5:** e1555.

29. Mizuhara H, O'Neill E, Seki N, Ogawa T, Kusunoki C, Otsuka K*, et al.* T cell activation-associated hepatic injury: mediation by tumor necrosis factors and protection by interleukin 6. *J Exp Med* 1994, **179**(5)**:** 1529-1537.

30. Gantner F, Leist M, Lohse AW, Germann PG, Tiegs G. Concanavalin A-induced T-cell-mediated hepatic injury in mice: the role of tumor necrosis factor. *Hepatology (Baltimore, Md)* 1995, **21**(1)**:** 190-198.

31. Van Antwerp DJ, Martin SJ, Kafri T, Green DR, Verma IM. Suppression of TNF-alpha-induced apoptosis by NF-kappaB. *Science* 1996, **274**(5288)**:** 787-789.

32. Liedtke C, Trautwein C. The role of TNF and Fas dependent signaling in animal models of inflammatory liver injury and liver cancer. *Eur J Cell Biol* 2012, **91**(6-7)**:** 582-589.

33. Kawasuji A, Hasegawa M, Horikawa M, Fujita T, Matsushita Y, Matsushita T*, et al.* L-selectin and intercellular adhesion molecule-1 regulate the development of Concanavalin A-induced liver injury. *J Leukoc Biol* 2006, **79**(4)**:** 696-705.

34. Wolf D, Hallmann R, Sass G, Sixt M, Kusters S, Fregien B*, et al.* TNF-alpha-induced expression of adhesion molecules in the liver is under the control of TNFR1--relevance for concanavalin A-induced hepatitis. *J Immunol* 2001, **166**(2)**:** 1300-1307.

35. Tiegs G, Wolter M, Wendel A. Tumor necrosis factor is a terminal mediator in galactosamine/endotoxin-induced hepatitis in mice. *Biochem Pharmacol* 1989, **38**(4)**:** 627-631.

36. Geisler F, Algül H, Paxian S, Schmid RM. Genetic Inactivation of RelA/p65 Sensitizes Adult Mouse Hepatocytes to TNF-induced Apoptosis In Vivo and In Vitro. *Gastroenterology* 2007, **132**(7)**:** 2489-2503.

37. Wroblewski R, Armaka M, Kondylis V, Pasparakis M, Walczak H, Mittrucker HW*, et al.* Opposing role of tumor necrosis factor receptor 1 signaling in T cell-mediated hepatitis and bacterial infection in mice. *Hepatology* 2016.

38. Festjens N, Vanden Berghe T, Cornelis S, Vandenabeele P. RIP1, a kinase on the crossroads of a cell's decision to live or die. *Cell Death Differ* 2007, **14**(3)**:** 400-410.

39. Kunstle G, Hentze H, Germann PG, Tiegs G, Meergans T, Wendel A. Concanavalin A hepatotoxicity in mice: tumor necrosis factor-mediated organ failure independent of caspase-3-like protease activation. *Hepatology* 1999, **30**(5)**:** 1241-1251.

40. Dondelinger Y, Aguileta MA, Goossens V, Dubuisson C, Grootjans S, Dejardin E*, et al.* RIPK3 contributes to TNFR1-mediated RIPK1 kinase-dependent apoptosis in conditions of cIAP1/2 depletion or TAK1 kinase inhibition. *Cell Death Differ* 2013, **20**(10)**:** 1381-1392.

41. Ting AT, Bertrand MJ. More to Life than NF-kappaB in TNFR1 Signaling. *Trends Immunol* 2016, **37**(8)**:** 535-545.

42. Liedtke C, Bangen JM, Freimuth J, Beraza N, Lambertz D, Cubero FJ*, et al.* Loss of caspase-8 protects mice against inflammation-related hepatocarcinogenesis but induces non-apoptotic liver injury. *Gastroenterology* 2011, **141**(6)**:** 2176-2187.

43. Vucur M, Reisinger F, Gautheron J, Janssen J, Roderburg C, Cardenas DV*, et al.* RIP3 inhibits inflammatory hepatocarcinogenesis but promotes cholestasis by controlling caspase-8- and JNK-dependent compensatory cell proliferation. *Cell Rep* 2013, **4**(4)**:** 776-790.

44. Kondylis V, Polykratis A, Ehlken H, Ochoa-Callejero L, Straub BK, Krishna-Subramanian S*, et al.* NEMO Prevents Steatohepatitis and Hepatocellular Carcinoma by Inhibiting RIPK1 Kinase Activity-Mediated Hepatocyte Apoptosis. *Cancer Cell* 2015, **28**(5)**:** 582-598.

45. Wong WW, Gentle IE, Nachbur U, Anderton H, Vaux DL, Silke J. RIPK1 is not essential for TNFR1-induced activation of NF-kappaB. *Cell Death Differ* 2010, **17**(3)**:** 482-487.

46. An J, Mehrhof F, Harms C, Lattig-Tunnemann G, Lee SL, Endres M*, et al.* ARC is a novel therapeutic approach against acetaminophen-induced hepatocellular necrosis. *J Hepatol* 2013, **58**(2)**:** 297-305.

47. Takemoto K, Hatano E, Iwaisako K, Takeiri M, Noma N, Ohmae S*, et al.* Necrostatin-1 protects against reactive oxygen species (ROS)-induced hepatotoxicity in acetaminophen-induced acute liver failure. *FEBS Open Bio* 2014, **4:** 777-787.

48. Zhang YF, He W, Zhang C, Liu XJ, Lu Y, Wang H*, et al.* Role of receptor interacting protein (RIP)1 on apoptosis-inducing factor-mediated necroptosis during acetaminophen-evoked acute liver failure in mice. *Toxicol Lett* 2014, **225**(3)**:** 445-453.

49. Dara L, Johnson H, Suda J, Win S, Gaarde W, Han D*, et al.* Receptor interacting protein kinase 1 mediates murine acetaminophen toxicity independent of the necrosome and not through necroptosis. *Hepatology* 2015, **62**(6)**:** 1847-1857.

50. Schneider AT, Gautheron J, Tacke F, Vucur M, Luedde T. Receptor Interacting Protein Kinase-1 (RIPK1) in hepatocytes does not mediate murine acetaminophen toxicity. *Hepatology* 2015.

51. Luedde T, Beraza N, Kotsikoris V, van Loo G, Nenci A, De Vos R*, et al.* Deletion of NEMO/IKKgamma in liver parenchymal cells causes steatohepatitis and hepatocellular carcinoma. *Cancer Cell* 2007, **11**(2)**:** 119-132.

52. Ehlken H, Krishna-Subramanian S, Ochoa-Callejero L, Kondylis V, Nadi NE, Straub BK*, et al.* Death receptor-independent FADD signalling triggers hepatitis and hepatocellular carcinoma in mice with liver parenchymal cell-specific NEMO knockout. *Cell Death Differ* 2014, **21**(11)**:** 1721-1732.

**Table 1. Sequence of primers used for qPCR**

| **Gene** | **Forward** | **Reverse** |
| --- | --- | --- |
| Mouse 18S | 5′-CGCCGCTAGAGGTGAAATTC-3′ | 5′-TTGGCAAATGCTTTCGCTC-3′ |
| Mouse TNFα | 5′-TAGCTCCCAGAAAAGCAAGC-3′ | 5′-TTTTCTGGAGGGAGATGTGG-3′ |
| Mouse IL-6 | 5′-CCGGAGAGGAGACTTCACAG-3’ | 5′-CAGAATTGCCATTGCACAAC-3′ |
| Mouse IFN-γ | 5’AGGTCAACAACCCACAGGTC3’ | 5’ATCAGCAGCGACTCCTTTTC3’ |
| Mouse-CXCL1 | 5’CGCCTATCGCCAATGAGC3’ | 5’GAACCAAGGGAGCTTCAGG3’ |
| Mouse TNFR1 | 5′-CAGAACACCGTGTGTAACTGC-3′ | 5′-GCA AGC GGA GGA GGT AGG-3′ |
| Mouse TNFR2 | 5′-CGC TGG TCT TCG AAC TGC-3′ | 5′-CAG GAG GAC ACT TAG CAC AGC-3′ |
| SAA1 | 5’-TGT TCA CGA GGC TTT CCA AG-3’ | 5’-GTC CTC TGC CGA AGA ATT CC-3’ |
| CCL20 | 5’-TCT GCT CTT CCT TGC TTT GG-3’ | 5’-TCA CCC AGT TCT GCT TTG G-3’ |
| Mouse-IKK-γ/NEMO | 5’-GGT GGA GAG ACT GAG CTT GG-3’ | 5’-CCT CTA AAG CTT GCC GAT CC-3’ |
